# The impact of gendered experiences on the career choice of swiss medical students: A qualitative study protocol

**DOI:** 10.1371/journal.pone.0302538

**Published:** 2024-05-20

**Authors:** Sylvie Arnoux, Laura E. Hirshfield, Milena Abbiati, Mathieu R. Nendaz, Georges L. Savoldelli, Nadia M. Bajwa

**Affiliations:** 1 Faculty of Medicine, Unit of Development and Research in Medical Education, University of Geneva, Geneva, Switzerland; 2 Department of Medical Education, University of Illinois College of Medicine, Chicago, Illinois, United States of America; 3 Department of Anaesthesiology, Division of Anaesthesiology, Pharmacology, Intensive Care and Emergency Medicine, Geneva University Hospitals, Geneva, Switzerland; 4 Department of Pediatrics, Division of General Pediatrics, Gynecology and Obstetrics, Geneva University Hospitals, Geneva, Switzerland; PLOS: Public Library of Science, UNITED KINGDOM

## Abstract

The problem of gender discrimination and sexual harassment in medicine is long-standing and widespread. This project aims to document and understand how gendered experiences encountered by final-year medical students in Switzerland are experienced by these individuals and how they influence their career choice. It also aims to identify representations and stereotypes linked to the different specialties. The project will take place at all Swiss universities offering a master’s degree in human medicine, for a total of 9 programs. Around 36 participants will be recruited. Semi-structured qualitative individual interviews will be conducted. Analysis will be based on Grounded Theory principles.

## Introduction

The problem of gender discrimination and sexual harassment in medicine is long-standing and widespread. Medical schools are more likely to be sources of discrimination and harassment than other science, technology, engineering, and mathematics (STEM) disciplines [1]. However, the reasons for these differences are not well-explained. Women faculty in medicine continue to experience discrimination and harassment in the form of unequal opportunities, lack of mentoring, lack of career advancement, lower pay, and lower job satisfaction [1,2]. In specialties such as surgery, women report the highest rates of gender discrimination and sexual harassment [1,3].

Most research thus far concerning gender discrimination and physicians has focused on women faculty and revealed that–compared to men faculty–they tend to have limited opportunities, lack of mentoring, slow career advancement, lower pay, and lower job satisfaction [2,4–6]. There are multiple factors that may explain this phenomenon: “the leaky pipeline”, tokenism, “the chilly climate”, and career-family balance [7]. These negative outcomes may dissuade women medical students from pursuing male-dominated specialties [5]. Men medical students may also be subject to discrimination when they consider entering female-dominated specialties [8,9].

The clinical clerkship has been reported to be the primary source of gender discrimination and sexual harassment for medical students, with over 90% of women and over 80% men reporting or observing discrimination or harassment during their clinical training [10]. At our own institution, the University of Geneva Faculty of Medicine, women medical students reported receiving gender biased remarks concerning their future career choice during their clinical clerkships at a higher rate than men medical students (84% vs 56%, P = .03) [3]. In addition to remarks, women may perceive that they are more often assigned administrative rather than clinical tasks in comparison to their male colleagues [11] Evaluations of women medical students may display bias by describing women based on their attitude or personal qualities instead of on their competence [12]. Men may also perceive gender discrimination in certain clerkships such as OB-GYN where they continue to feel that their final clerkship evaluation is negatively affected by their gender [8,9].

A majority of those who have faced discrimination and harassment do not report their experiences because of the arduousness of the process, because they assume that no action will be taken against the offending party, and out of fear of receiving a negative clerkship evaluation [11,13]. Some students go to great lengths to avoid future discrimination and harassment by changing services, changing curricular tracks, stopping class attendance, or changing their desired career choice altogether [11,14]. When students perceive that gender discrimination and sexual harassment make up the hidden curriculum of the institution, they may undergo a process of acculturation in order to protect themselves [4,11,14]. When women struggle to define their role in the team they may default to stereotypical gender roles [15]. They begin to identify and integrate those behaviors that are demonstrated and considered acceptable by other female physicians such as being serious, proving themselves, and avoiding discussion of their personal lives. This phenomenon is described as the “gender learning curve” and helps women medical students to reconcile their future identity as a women physician [15]. They quickly identify the hospital environment as one that values traditionally male characteristics of objectivity, authority, detachment, and competition [14]. Students ultimately start to normalize and expect these experiences in the future. For example, while illegal, during the residency application process women are frequently posed questions concerning family planning and personal matters so much so that some medical schools provide courses to medical students on how to handle these inappropriate questions [16]. Women are also more likely than men to let these negative experiences dissuade them from their initial career choice [10].

The purpose of this study is to explore how gendered experiences in the clinical clerkships, encountered by Swiss medical students, may influence and ultimately affect their future specialty career choice. The results may help us to better understand the effect of gendered experiences on student career choice motivations and may inform future strategies to support students during their clerkship training and during their career choice decision-making process.

## Methods

### Conceptual frameworks

We will use the following sensitizing concepts to guide our study: *gender discrimination*, *sexual harassment*, *and gendered experiences*. Harassment refers to unsolicited behavior ranging from remarks or comments to advances. Gender discrimination includes differences in salaries, career advancement and opportunities, and differences of treatments in the daily interactions such as different expectations, stereotyping, and gendered expectations [15,17,18]. Hill and Vaughan describe a decisional process through gendered experiences where students negotiate their paradigmatic trajectory [19]. Students first “see, hear, and do” before advancing to a stage of “imagining” where they can see themselves in the profession before arriving to their ultimate career choice [19]. The first stages of “seeing, hearing, and doing” involve varied experiences and observations where the student is confronted with how well they fit with the profession, what others communicate about the profession, and how many meaningful clinical experiences they accumulate through their clinical clerkship [19]. When students do not go through this developmental and socialization process of professional identity formation, they may end up dis-identifying and avoiding the specialty in question [14,20].

Social identity theory, intersectionality, and critical theory are important conceptual frameworks when dealing with gender issues. According to the social identity approach, individuals’ sense of self is influenced by the social groups to which they feel that they belong, termed the in-groups [21]. Even preconceived notions of an imperfect fit may dissuade women from joining predominantly male professions [22]. Using a social identity approach, we can understand why and through what mechanisms academic medicine continues to create a “culture of exclusion” for women in order to maintain the in-group and distance the out-group [23].

When investigating the effects of gender, it would be a miscalculation to ignore the intersection of other social identities (origin, race, sexuality) when examining gender discrimination and sexual harassment. Intersectionality refers to the ways in which different social identities interact to shape an individual’s experience; “recognizing the impossibility of separating social categories such as race, class, gender, and sexuality: the multiple identities we possess should be seen as transformational rather than additional” [24]. Considering the multiple social identities held by medical trainees and professionals [25–27], we hypothesize that intersectionality can deepen our understanding of biases and inequities in the context of complex intergroup relations and provide a different lens through which to look for ways to mitigate biases.

Finally, in order to deconstruct assumptions about gender discrimination in medicine, it is useful to take into consideration the social context and power hierarchies that may contribute to these intergroup conflicts anchored in our medical students’ everyday lives. Critical theory [28,29] which relies on social, political, cultural, economic, ethnic, and gender contexts to frame reality and to understand how institutional and cultural forces contribute to a phenomenon will help to frame our research questions and to interpret the insights gathered from our questioning of medical students on their experiences. The knowledge gathered is transactional and subjective relying heavily upon the values of the research team and the interpretation of the data [30]. Using a critical theory lens permits the participants in this study to partake in counter-story telling [31] that may bring insights into how gender discrimination in all forms (microaggressions, gendered experiences, and sexual harassment) influence career choice. The ultimate goal being a critique of the status quo and a generation of strategies to oppose experiences of gender discrimination.

### Duration of the project

The study is expected to last 18 months (01.2024–06.2025).

### Study design

We intend to conduct a qualitative analysis of individual semi-structured interviews using a Grounded Theory approach, specifically constant comparative analysis [32]. Individual interviews were deemed to be appropriate for our study because they will permit participants to share personal and confidential experiences that they may be unwilling to share in a group setting. The interview format will also allow us to generate rich insight into the phenomenon being studied.

### Sampling

The study will be conducted at the following Swiss faculties of medicine: Geneva, Basel, Bern, Fribourg, Lausanne, Lugano, and Zurich (including St. Gallen and Lucerne campuses). In Switzerland, students complete a 6-year curriculum. The Bachelor’s years, the first 3 years of the curriculum, focus on basic medical sciences and an introduction to the clinical sciences, whereas the Master’s years, last 3 years, focus on clinical immersion.

Starting in March 2024, 30 to 40 medical students will be recruited among the nine medical master’s program for an aim of 36 participants. This number seems appropriated as it is both feasible and high enough to provide a sample as diverse as possible [33,34]. An email with the recruitment criteria will be sent to medical students, and snowball sampling will be used to complete the recruitment. The number of participants will be adapted accordingly when data sufficiency has been reached [33]. Participants will be able to choose between a face-to-face or remote interview. The Zoom video-conferencing service (Zoom Videos Communications Inc., San Jose, United States of America) will be used if the second option is chosen. The interview will be recorded on the personal computer of the interviewer (not on the cloud). All students will be compensated (50 CHF) for each individual interview.

### Ethical considerations

This project obtained the approval of the CUREG ethics committee of the University of Geneva (protocol number: CUREG-20231106-292-2). Written consent will be signed by participants and stored in academic servers. In our study, we will use sensitive private information provided by human participants. Therefore, special care will be taken to handle security and anonymization of sensitive data. Sensitive data transfers will be end-to-end encrypted and encryption keys will be managed only by authorized employees. Each participant will be given a code number and transcriptions of interviews will be anonymized. The audio files and the correspondence list will be destroyed.

Participants relating important episodes of discrimination will be encouraged to contact the office of student affairs to seek support and counseling. A document listing contacts and resources will be provided if required.

### Data source and data collection

Prior to the main data collection, two pilot interviews will be conducted with medical students, one man and one woman. They will be informed of the testing nature of the interviews. These pilots will inform the research team on the relevance and quality of the interview guide.

### Data collection tool

We anticipate that the interviews will last from 45 minutes to 1 hour. Critical theory informed the creation of our interview guide to explore and challenge models currently used to explain gender discrimination (e.g., role congruity theory, lack of fit) in order to better understand why there is a gender divide concerning medical student career choice. Participants will be questioned about their career choice motivations before broaching more sensitive topics of gender discrimination; see Appendix 1 for the interview guide. We expect our interview guide to evolve during the data collection process as in-depth interviews will generate new insight and aspects that will be further investigated [32,33].

### Data sufficiency

Recruitment will stop when sampling objectives are met. Furthermore, special attention will be paid to the sufficiency of the data gathered. According to Grounded Theory principles, at least 20 interviews must be conducted to gather enough data and build a theory [32,35]. This number will also allow us to reach sufficient level of information power given the aim of our study and the expected quality of dialogue [34]. Sufficiency will be reached when the data will offer a deep and large enough understanding of the participants’ experiences [33,34]. We estimate that we will have recruited enough participants by the end of June 2024.

### Data analysis approach

An inductive approach will be used in the analysis. The inductive approach will be based on constructivist Grounded Theory [32]. The method of constant comparative analysis will be used to compare data of participants for similarities and differences [32].

### Data analysis process

In our analysis, two authors (SA and NB) will carry out an ongoing analysis of interview transcripts to identify themes. SA and NB will meet to compare themes and discuss differences in identification of themes to enrich understanding and reflexivity. 5 transcripts will then be independently line-by-line coded by SA and NB using the list of themes previously discussed. SA, NB, GS, MA and LH will meet once this step is completed to discuss the codes and organize them in a codebook. This structure will then be used to code the rest of the transcripts. Any emerging code will be discussed and added to the codebook to refine the comprehension and analysis of the data. Scientific software, ATLAS.ti 5.0 (Berlin), will be used to assist with data management and retrieval.

Major themes and subthemes will be identified inductively and iteratively and definitions for these themes will be determined. We will then compare the data collected with our initial themes and label the data with codes that were identified for each subtheme. To enhance our own sensitivity during the analysis, and to identify our own biases, an ongoing team discussion will be conducted.

Memo-writing will be used throughout to deepen analysis and document thoughts regarding the identification of themes within the ‘theoretical coding’ stage of analysis. The memos will include informal information on the context, nonverbal behavior of the participants, emotionality, and general reactions to the questions posed. Regular meetings will be used to discuss when theoretical sufficiency has been reached. Finally, the themes will be organized into a conceptual model.

Due to the cultural context of Switzerland, interviews will be conducted in one of three official Swiss languages (French, German, and Italian). One member of the research team is fluent in Italian and will conduct the interviews with the students from the medical school in Lugano. Interviews at the universities of Geneva, Fribourg, and Lausanne will be conducted in French. French is the mother tongue of the researcher in charge of the rest of the data collection. She is also fluent in German, which is the chosen language to conduct the interviews at the universities of Bern, Basel, Zurich, St. Gallen, and Lucerne [36]. However, the mother tongue of participants at the German speaking universities is a dialect of German, Swiss German. As none of the researchers speak this dialect, the interviews will be conducted either in German, English or French. Therefore, as none of these languages are used by the participants to express themselves in their daily lives, we might encounter a loss of details, reflexivity, and depth during those interviews. To preserve the integrity of data, interviews will be transcribed in their original language. Translation will occur after the transcriptions. While translating, special attention will be paid to preserve the meaning attributed by participants to the words and their use. Memos will provide useful context information to verify the accuracy of the translated transcripts [37–39]. Regular meetings will be held to discuss the coding and external advice will be sought if difficulties related to the languages are identified.

### Reflexivity

We are cognizant that the make-up of our research team will have an impact on our qualitative study design, data collection, data analysis, and interpretation of our results. Thus, our goal was to bring together a team that is familiar with medical education but that comes from different disciplines related to the field. The lead author (NB) is a female pediatrician and a pediatric residency program director. The other authors will all be specialists in medical education: a male internist (MN), a male anesthesiologist (GS), two female sociologists (SA and LH), and a female psychologist (MA). We hope that creating this balanced team with differing backgrounds will bring richness and insight to our data analysis and interpretation of results. Because some of our roles in our institution may create a power differential with our participants, a doctoral student in medical education (SA) and a psychologist (MA) will conduct the interviews.

The team will meet on a regular basis to discuss the coding process and the analyses to recognize and avoid biases. Consensus will be sought among the team members regarding the categories. LH will be included in the coding process. As a gender studies specialist, she will bring a deeper understanding analysis of the data. Furthermore, these meetings will give researchers the opportunity to reflect on the research process and the positionality of the team members.

Throughout the study, an audit trail involving information of all decisions made will be documented. This will provide transparency regarding each step, from initial conceptualization to reporting. It will also include raw data, and the team’s iterative analyses and reflections, will be documented to enhance the trustworthiness of results.

## Expected results

We expect that our results will include a rich discourse of experiences and motivations regarding medical students’ career choice. Men are expected to report less occurrences of gendered experiences as the literature shows that they are less likely to be exposed to such experiences. However, we expect them to share testimonies and stories about gendered experiences lived by colleagues during their clerkships. Data relating episodes of discrimination or sexual harassment will also be collected. A specific procedure has been detailed previously to offer help to participants relating those events.

### Strengths

This study will investigate the influence of gendered experiences on medical students’ career choice. Qualitative methods will offer rich and deep information regarding the motivations and experiences of students. Our study is unique in that this is a national study involving all the medical faculties in Switzerland and that we expect to have a diverse panel of participants. These methods are seldom used to understand the process of selecting a career and will therefore offer rich insight on how gendered experiences such as discrimination or sexual harassment are lived and experienced by medical students. The results may show how these events, or their anticipation can influence the choice of elective clerkship and the career choice of medical students. Based on the results, recommendations for curricular change could also be generated to limit discriminatory experiences and to address the gender norms perpetuated by faculty during the clinical clerkships. This study could also inform on the development of educational strategies to help students with their career choice decision-making process.

### Limitations

For practical reasons, the recruitment method will generate a self-selected sample which will cause a selection bias as participants may have a prior interest in the topic. Participants may also show various levels of sensitivity regarding gender, discrimination, or harassment, which may induce differences in the quality and depth of responses to interview questions. Prompts have been added to the interview material to help participants recount their experiences and related feelings and emotions. Men participants may also feel less comfortable relating gendered experiences to the two female interviewers. The use of three languages may result in a loss of detail or meaning in the coding process as the subtleties of language differences may go unnoticed.

## Supporting information

S1 File Interview guide. (DOCX)
