# Supplementary material for: The impact of gendered experiences on the career choice of swiss medical students: A qualitative study protocol
Source: PLoS One. 2024 May 20;19(5):e0302538. doi: 10.1371/journal.pone.0302538 (PMC11104654; doi:10.1371/journal.pone.0302538)
Supplement: S1 File — (DOCX) [file pone.0302538.s001.docx]

**S1 Interview guide**

Introduction: Presentation; Purpose of the study; confidentiality; recording

| Getting to know you | - Can you briefly present yourself? (Age, origins, …) - Can you tell me a little bit about what your future career plans are? Have you decided on a specific specialty? What kind of clerkships have you participated in (*which year, which place*), for how long ? - If so, what is it about this specialty that draws you to it? (skills, income, work-life balance…) - Can you tell me a little bit about your experiences during the other clerkships? What was it about these clerkships that made you think that it wouldn’t be a good fit for you going further? - If undecided, what specialties seem the most attractive to you right now? *Ask the most likely* |
| --- | --- |
|  | *If the participant hesitates between two specialties: for each questions in box 2, ask about both specialties*  *If the participant hasn’t done her/his clerkship in the desired specialty yet, read the question signaled by ***  *Questions are at first referring to the specialty chosen for the internship. Participants are invited in a second time to refer to the specialties they haven’t chosen* |
| Experiences during the clerkships | - What are your perceptions about this specialty? Is there any rumor or common knowledge? (**Add** the fit for a man or a woman). What stereotype do you have about this specialty choice? Have you heard about stereotypes in other specialties? Did those stereotypes influence your choice? - When choosing, did you talk about it with someone? Did you seek counsel? From whom? - Did you find role models during the clerkship? Can you describe her/him and why she/he is a role model for you (negative or positive)? And during the clerkship in other specialties?   **Have you already met role models practicing this specialty? Do you expect to meet one? What characteristics are important for you when looking for a role model?   - Were your supervisors mainly men or women? - Do you feel that supervisors of a certain gender are lacking in this specialty? - What is your feeling about men and women having an equal chance to enter into this specialty? Do you identify any facilitators or obstacles? - barriers are identified, how do you feel about having to adjust to overcome these barriers? - Did you receive any remarks during these clerkships pointing out to you that it would be difficult for you to pursue this specialty? **Add** because of your gender? If so, what was the nature of these remarks? Did these remarks influence your ultimate career choice? - How did this specific clerkship go for you? Did you experience some discrimination based on your gender during the evaluation of this clerkship? And have you experienced this type of discrimination in other clerkships?   **What kind of experiences and exposures do you expect during this clerkship? Are you expecting any forms of discrimination? Why?   - What do you think the impact of the gender has been, if any, on your experience during the clerkship? What about other clerkships?   **What do you think the impact of gender will be, if any, on your experience during the clerkship?   - Do you feel that there are any advantages of being of your gender in this field? And in others? - Were there times where your access to patients was limited because of your gender?   **Do you expect facing some difficulties to access the patients during the clerkship?   - Did you notice differences in how the other gender was treated during the clerkship in comparison to your own gender? - Did you notice differences in interactions with patients? With staff (nurses)? With supervisors? In what way? - Do you think some members of the multi-disciplinary team interact with you differently because of your gender? - If yes, in what way? And which professional and why do you think this is the case? - And in other clerkships?   **Do you anticipate any differences in how the other gender will be treated during the clerkship in comparison to your own gender?   - Would you feel comfortable challenging interactions where you thought there was gender bias? If not, why not? Who could ? - Besides gender, are there other characteristics (physical appearance, origin, or sexual orientation) that changed the way that you were treated during your clerkship (intersectionality)?   **Do you anticipate that characteristics other that your gender may change the way you will be treated during your clerkship?   - If you have already applied for an internship, have you felt any form of discrimination during the process? |

Conclusion: Questions ? Anything to add ?

Acknowledgements; availability of the results; consent form
